# Complete genome sequences of 12 lytic phages against multidrug-resistant *Enterococcus faecalis*

**DOI:** 10.1128/mra.00687-24

**Published:** 2024-09-10

**Authors:** Oumarou Soro, Collins Kigen, Andrew Nyerere, Moses Gachoya, James Wachira, Vanessa Onyonyi, Martin Georges, Allan Wataka, Stephen Ondolo, Karen Cherono, Erick Odoyo, Lillian Musila

**Affiliations:** 1 Department of Molecular Biology and Biotechnology, Pan African University Institute for Basic Sciences, Technology, and Innovation, Nairobi, Kenya; 2 Department of Emerging Infectious Diseases, Walter Reed Army Institute of Research-Africa, Kericho, Kenya; 3 Department of Medical Microbiology, Jomo Kenyatta University of Agriculture and Technology, Nairobi, Kenya; Loyola University, Chicago, Illinois, USA

**Keywords:** bacteriophage therapy, *Enterococcus faecalis*, complete genome, Kenya

## Abstract

We report the genome sequences of 12 *Enterococcus faecalis* phages isolated in Kenya, belonging to the genus *Copernicusvirus*, *Efquatrovirus*, *Saphexavirus*, and *Kochikohdavirus*. They have double-stranded DNA with lengths varying from 17,979 to 147,374 bp and G+C content from 33.14% to 40.05%. The genomes contain 28–250 coding sequences.

## ANNOUNCEMENT

Nosocomial infections caused by antibiotic-resistant *Enterococcus faecalis* have increased, endangering public health (1). Due to the declining effectiveness of antibiotics for treating enterococcus-related infections, alternative therapies such as bacteriophages (phages) are being considered (2, 3). We report whole-genome sequences of 12 *E. faecalis* phages isolated in Kenya.

Phages were isolated from wastewater treatment plant samples collected in Nairobi, Kenya, through an enrichment method using clinical isolates of multidrug-resistant *E. faecalis* as hosts (4) (Approval #WRAIR-2089/KEMRI-2767), as described elsewhere with slight modifications (5). Briefly, 50 mL of wastewater was centrifuged (12,000 × *g*, 10 min), and the supernatant was filtered (0.22 µm). Eight milliliters of the supernatant was mixed with 2 mL of 5× tryptic soy broth (TSB; Oxoid Ltd., Basingstoke, England) and 50 µL of bacterial culture grown in 1× TSB (37°C, 200 rpm, 24 h). The mixture was incubated (37°C, 200 rpm, 24 h), and the bacterial debris was eliminated through centrifugation. Phages were isolated using plaque assays (6) and purified by three rounds of single plaque isolation. Pure phages were amplified on the enrichment strain in 50 mL TSB and concentrated by centrifugation (10,000 × *g*, 18 h) to reach a titer of >10^10^ PFU/mL for DNA extraction (7). Host RNA and DNA were removed using RNase A and DNase I (ThermoFisher Scientific, USA), deproteinization using Proteinase K at 56°C for 1 h 30 min (5), and phage DNA isolated using a Norgen phage DNA isolation kit (Norgen Biotek Corp., ON, Canada). Sequencing libraries were prepared using the Collibri PCR-free ES DNA library preparation kit (Invitrogen, California, USA) and sequenced on an Illumina MiSeq instrument using a 600-cycle MiSeq reagent kit v3, yielding 350-bp maximum paired-end reads. The raw reads were quality-checked using FastQC v0.12.1 (8), trimmed with fastp 0.23.4 (9), and assembled with shovill 1.1.0 (https://github.com/tseemann/shovill). After assembly, QUAST 5.2.0 (10) was used to perform a quality check. CheckV v1.0.3 (11) and Bandage 0.8.1 (12) were used for genome completeness prediction (100%) and assembly visualization, respectively. Pharokka v1.5.1 (13) was used for genome annotation with Phanotate 1.5.1 (14) to identify coding sequences and screen for antimicrobial resistance (AMR) and virulence genes against the Comprehensive Antibiotic Resistance Database (15) and Virulence Factor Database (16), respectively. Taxonomic classification was performed using Mash alignment against the INPHARED database (17). The lifecycle was determined by PhageLeads (18), and termini and packaging mechanisms were identified by PhageTerm version 1.0.11 (19) using both the raw reads and the assembled genomes. ARAGORN v1.2.41 (20) and tRNAscan-SE v2.0.12 (21) were used to predict tRNA genes. Default parameters were used for all software tools.

The phages belonged to group I of Baltimore’s classification (22) and varied in length from 17,979 to 147,374 bp, with G+C content ranging from 33.14% to 40.05%. The genomes contained 28–250 coding sequences and 0–8 tRNAs (Table 1). The phage families, genera, and termini results are presented in Table 1. The genomes lacked predicted AMR, virulence, and lysogeny genes, suggesting a strictly virulent lifecycle and promising candidates for phage therapy.

**TABLE 1:** Genomic characteristics of the 12 *E. faecalis* phages^*a*^

Phage name	Bacterial host ST	Sample collection site (GPS coordinates)	Family	Genus	Genome length (bp)	G+C content (%)	No. of CDSs	Genome coverage (×)	No. of raw reads	No. of tRNA	Termini	GenBank accession no.	SRA accession no.
Enterococcus phage vB_Efs1_KEN01	6	Ruai sewage treatment plant (1°14′26.6″S 37°01′01.9″E)	*Rountreeviridae*	*Copernicusvirus*	17,979	33.14	28	119.32	424,928	0	Unknown	PP582177	SRR28445062
Enterococcus phage vB_Efs4_KEN02	947	Ruai sewage treatment plant (1°14′26.6″S 37°01′01.9″E)	Unclassified	*Efquatrovirus*	39,740	34.8	71	60.58	548,194	0	Cos (3′)	PP582178	SRR28445061
Enterococcus phage vB_Efs6_KEN03	6	Ruai sewage treatment plant (1°14′26.6″S 37°01′01.9″E)	Unclassified	*Saphexavirus*	57,731	40.03	117	112.38	275,057	2	DTR (short)	PP582179	SRR28445058
Enterococcus phage vB_Efs8_KEN04	1,904	Ruai sewage treatment plant (1°14′26.6″S 37°01′01.9″E)	*Herelleviridae*	*Kochikohdavirus*	142,402	36.01	238	67.58	260,290	8	DTR (long)	PP582180	SRR28445057
Enterococcus phage vB_Efs10_KEN05	6	Ruai sewage treatment plant (1°14′26.6″S 37°01′01.9″E)	Unclassified	*Saphexavirus*	59,225	39.85	119	84.18	276,348	1	DTR (short)	PP582181	SRR28445056
Enterococcus phage vB_Efs19_KEN07	1,907	Ruai sewage treatment plant (1°14′26.6″S 37°01′01.9″E)	Unclassified	*Efquatrovirus*	40,417	34.9	73	66.83	210,284	0	Cos (3′)	PP582182	SRR28445055
Enterococcus phage vB_Efs22_KEN09	1,908	Ruai sewage treatment plant (1°14′26.6″S 37°01′01.9″E)	Unclassified	*Efquatrovirus*	41,162	34.76	76	68.35	172,547	0	DTR (short)	PP582183	SRR28445054
Enterococcus phage vB_Efs25_KEN11	6	Ruai sewage treatment plant (1°14′26.6″S 37°01′01.9″E)	*Herelleviridae*	*Kochikohdavirus*	144,458	35.9	241	64.26	368,696	0	DTR (long)	PP582184	SRR28445053
Enterococcus phage vB_Efs26_KEN12	6	Ruai sewage treatment plant (1°14′26.6″S 37°01′01.9″E)	*Rountreeviridae*	*Copernicusvirus*	18,044	33.25	28	72.53	337,960	0	Unknown	PP582185	SRR28445052
Enterococcus phage vB_Efs30_KEN14	28	Ruai sewage treatment plant (1°14′26.6″S 37°01′01.9″E)	Unclassified	*Efquatrovirus*	41,198	34.89	75	67.21	237,448	0	Cos (3′)	PP582186	SRR28445051
Enterococcus phage vB_Efs6_KEN16	6	JKUAT water dam (1°05′32.2″S 37°01′03.5″E)	*Herelleviridae*	*Kochikohdavirus*	147,374	35.94	250	68.42	306,288	7	DTR (long)	PP582187	SRR28445060
Enterococcus phage vB_Efs19_KEN17	1,907	JKUAT water dam (1°05′32.2″S 37°01′03.5″E)	Unclassified	*Saphexavirus*	57,031	40.05	115	68.60	323,139	1	DTR (short)	PP582188	SRR28445059

^*a*^ Cos (3′), 3′ cohesive sequence; DTR, direct terminal repeats; CDSs, coding sequences; SRA, Sequence Read Archive; JKUAT, Jomo Kenyatta University of Agriculture and Technology; ST, sequence type.

## Data Availability

The genome sequences of the 12 *E. faecalis* phages are available in GenBank and Sequence Read Archive under the accession numbers listed in Table 1.
